# Nicotinamide N-Methyltransferase Is a Prognostic Biomarker and Correlated With Immune Infiltrates in Gastric Cancer

**DOI:** 10.3389/fgene.2020.580299

**Published:** 2020-10-28

**Authors:** Miaowei Wu, Weilei Hu, Guosheng Wang, Yihan Yao, Xiao-Fang Yu

**Affiliations:** ^1^Cancer Institute, Second Affiliated Hospital, Cancer Institute, Zhejiang University School of Medicine, Hangzhou, China; ^2^Institute of Translational Medicine, Zhejiang University School of Medicine, Hangzhou, China; ^3^Department of Surgical Oncology, Second Affiliated Hospital, Zhejiang University School of Medicine, Hangzhou, China

**Keywords:** NNMT, gastric cancer, immune infiltration, treatment, biomarker

## Abstract

Gastric cancer (GC) is the third most common cause of cancer-related death in the word. Immunotherapy is a promising treatment of cancer. However, it is unclear which GC subpopulation would benefit most from immunotherapy and it is necessary to develop effective biomarkers for predicting immunotherapy response. Nicotinamide N-methyltransferase (NNMT) is a metabolic regulator of cancer-associated fibroblast (CAF) differentiation and cancer progression. In this study, we explored the correlations of NNMT to tumor-infiltrating immune cells (TIICs) and immune marker sets in The Cancer Genome Atlas Stomach Adenocarcinoma STAD (TCGA-STAD). Subsequently, we screened the NNMT correlated genes and performed the enrichment analysis of these genes. We eventually predicted the 19 most potential small-molecule drugs using the connectivity map (CMap) and Comparative Toxicogenomics Database (CTD). Also, nadolol, tranexamic acid, felbinac and dapsone were considered the four most promising drugs for GC. In summary, NNMT can be used as a prognostic biomarker that reflect immune infiltration level and a novel therapeutic target in GC.

## Introduction

Gastric cancer (GC) is the third leading cause of death by cancer worldwide (Bray et al., 2018). Although the prevalence and death rates of GC have deceased in recent years, thanks to the eradication of *Helicobacter pylori*, GC is still a significant health risk, and the age-standardized 5-year net survival is <30% in most countries (Allemani et al., 2018). The disease is often diagnosed during its advanced stages, leading to the high mortality for GC patients (Digklia and Wagner, 2016). Hence, knowing more about the molecular mechanisms contributing to GC progression can help us develop more effective treatment regimens.

As we have come to appreciate the importance of tumor-associated immune mechanisms in GC, immunotherapy has become a standard of treatment for many solid tumors (Larkin et al., 2019; Mok et al., 2019), but not yet for gastrointestinal cancers (Pagni et al., 2019). Although immune checkpoint blockade with antibodies targeting cytotoxic CTLA-4, PD-1, and PD-L1 has shown clinical activity in some GC patients, it is still unclear which GC subpopulation would benefit most from checkpoint inhibitors (Magalhaes et al., 2018), since PD-L1 immunohistochemistry and tumor mutational burden remain inadequate indicators for guiding treatment (Pagni et al., 2019). Thus, biomarkers for predicting outcome and immunotherapy response are clearly needed for GC patients.

Nicotinamide N-methyltransferase (NNMT), originally known as a vitamin B3 clearance enzyme, is a key metabolic enzyme that catalyzes the N-methylation of nicotinamide (NAM, one of the forms of vitamin B3) and other pyridines (Alston and Abeles, 1988), using the universal methyl donor S-adenosyl methionine (SAM) (Pissios, 2017). In the 1950s, Gulio Cantoni partially purified NNMT and raised that ATP and methionine form a high-energy intermediate used for the methylation of NAM (Cantoni, 1951). N1-methylnicotinamide, a product of NNMT, can be further oxidized into N1-methyl-2-pyridone-5-carboxamide and N1-methyl-4-pyridone-3-carboxamide by aldehyde oxidase, then these metabolites are finally excreted in the urine (Felsted and Chaykin, 1967; Aksoy et al., 1994). Thus, NNMT was classified as a detoxification enzyme since methylation is a detoxification pathway. In 1990s, Aksoy and Yan cloned human and mouse NNMT genes (Aksoy et al., 1994; Yan et al., 1997). The canonical NNMT gene structure is composed of three exons and two introns. The NNMT protein is well conserved in mammals, with 85% sequence similarity between humans and mice (Schmeisser et al., 2013).

In recent years, role of NNMT has been expanded beyond excess vitamin B3 clearance as it involves in regulation of multiple metabolic pathways in adipose, liver, and cancer cells through consuming and generating active metabolites (Pissios, 2017). Notably, high NNMT expression has been observed in many solid tumors and identified as a marker of malignant degree (Li et al., 2018); these tumors include breast cancer (Wang et al., 2019), renal cell carcinoma (Sartini et al., 2006; Holstein et al., 2019), bladder cancer (Sartini et al., 2013b), non-small cell lung cancer (Sartini et al., 2013a), oral squamous cell carcinoma (OSCC) (Emanuelli et al., 2010; Sartini et al., 2012), skin cancer (Ganzetti et al., 2018; Pompei et al., 2019), esophageal squamous cell carcinoma (Cui et al., 2019), and ovarian cancer (Eckert et al., 2019). In addition, NNMT overexpression was found to be associated with increased tumorigenesis of colorectal cancer (Xie et al., 2014), bladder cancer (Wu et al., 2008), squamous cell carcinoma (Hah et al., 2019), and clear cell renal cell carcinoma (Tang et al., 2011). Consistently, NNMT silencing is linked with apoptosis of breast cancer cells (Zhang et al., 2014), as well as the decreased tumorigenicity of non-small cell lung cancer cells (Sartini et al., 2015), oral carcinoma cells (Pozzi et al., 2013), and glioblastoma cells (Palanichamy et al., 2017). However, the primary functions and mechanisms of NNMT in tumor development and tumor immunology are not fully understood.

In the present study, we comprehensively evaluated the correlation of NNMT expression with prognosis in GC patients with various clinicopathological factors, together with the potential functions of NNMT and its correlated genes in GC. Moreover, we explored the effect of NNMT on immune infiltration in GC. Finally, we predicted the usefulness of existing drugs in potentially targeting NNMT and its correlated genes.

## Materials and Methods

### Oncomine Analysis

To compare the NNMT mRNA expression patterns of tumor tissue and normal tissue, we used the Oncomine database^1^ (Rhodes et al., 2007). This database is an online cancer microarray database containing 715 gene expression data sets and data from 86,733 cancerous and normal tissues. The expression of NNMT mRNA in GC specimens was compared to that in normal controls, using a Student’s *t*-test to generate a *p*-Value.

### Gene Expression Profiling Interactive Analysis (GEPIA) Dataset

This database is an interactive web server for analyzing the RNA sequencing expression data from The Cancer Genome Atlas (TCGA) projects. GEPIA is an interactive web server for analyzing the RNA sequencing expression data from the TCGA and GTEx projects, using a standard processing pipeline. GEPIA provides key interactive and customizable functions, including differential expression analysis, patient survival analysis, detection of similar genes, correlation analysis, and profiling plotting (Tang et al., 2017). Survival curves and gene expression correlation were analyzed via GEPIA. The Spearman method was used to determine the correlation coefficient. Tumor and normal tissue datasets were used for the analysis.

### Kaplan-Meier Plotter

The prognostic value of NNMT mRNA expression was explored using an online database, Kaplan-Meier Plotter^2^ (Szasz et al., 2016), which contained gene expression data and survival information of GC patients. To analyze the overall survival (OS), progression-free survival (FP), and post-progression survival (PPS) in patients with GC, patients were split into two groups by median expression (high vs. low expression) and assessed by a Kaplan-Meier survival plot, with the hazard ratio with 95% confidence intervals and logrank *p-*Value.

### cBioPortal Analysis

The cBioPortal for Cancer Genomics^3^ is a Web resource for exploring, visualizing, and analyzing multidimensional cancer genomics data (Gao et al., 2013). We used cBioPortal to inquire into the genes related to NNMT in stomach adenocarcinoma (STAD).

### LinkedOmics Analysis

The LinkedOmics database^4^ is a web-based platform for analyzing 32 cancer types and a total of 11,158 samples from TCGA project (Vasaikar et al., 2018). We used LinkedOmics to investigate NNMT expression levels in STAD patients with different stages.

### Protein–Protein Interaction (PPI) Network Construction

The NNMT co-expressed genes were first mapped to the Search Tool for the Retrieval of Interacting Genes/Proteins (STRING) database^5^ (Szklarczyk et al., 2017) to assess the functional associations among the target genes. Only interactions with a combined score of >0.4 were considered significant. Next, to obtain the hub genes, the degree of connectivity in the PPI network was analyzed and the PPI network was visualized using Cytoscape software (version 3.6.0).

### Gene Correlation Analysis

The lymphocyte-specific immune recruitment (LYM) metagene signature was defined by the average of the expression levels of PTPRC (CD45), CD53, LCP2 (SLP-76), LAPTM5, DOCK2, IL10RA, CYBB, CD48, ITGB2 (LFA-1), and EVI2B (Cheng et al., 2013). Correlation analysis of NNMT expression and LYM metagene was performed on TCGA expression data.

### ESTIMATE Database Analysis

Immune and stromal scores were calculated from Estimation of STromal and Immune cells in MAlignant Tumors using Expression (ESTIMATE) data^6^. ESTIMATE is an algorithm providing scores for the levels of immune cell infiltration and stromal tissue in tumor tissues. The “ESTIMATE score” was calculated by combining the immune and stromal scores.

### Evaluating the Immune Response of 22 Tumor-Infiltrating Immune Cells (TIICs) in STAD via CIBERSORT

CIBERSORT^7^, a method for characterizing cell composition of complex tissues from their gene expression profiles (Newman et al., 2015). Densities of TIICs can therefore be precisely assessed via this method. Our current analysis measured the immune response of 22 TIICs in STAD via CIBERSORT, gene expression datasets were set out using standard annotation files and uploaded to CIBERSORT web portal. To estimate the influence of NNMT expression, we divided STAD samples into low-expression and high-expression based on the median value of the NNMT expression, and then used the data to make violin plots.

### Tumor Immune Estimation Resource (TIMER) Database Analysis

Tumor immune estimation resource is a broad database for analyzing immune infiltration in various cancers^8^ (Li et al., 2017). TIMER facilitates the computation of the association between densities of TIICs and NNMT gene expression based on a deconvolution of a previously published statistical method. The correlations between NNMT expression and checkpoint molecules was calculated. The Correlation module was used to draw the expression scatterplots comparing a pair of user-defined genes in a given cancer type, together with the Spearman’s correlation and estimated statistical significance. NNMT was represented on the y-axis with gene symbols, whereas the correlated marker genes were placed on the x-axis. The gene expression levels were determined with log2 RSEM. Options used for partial correlation conditioned on tumor purity or age were also used.

### Prediction of Novel Drugs for GC

The Connectivity Map (CMap) (Subramanian et al., 2017) is a platform for finding connections among small molecules that share the same mechanism of action and associations with chemicals and physiological processes, diseases, and drugs. In the present study, the NNMT-correlated genes were divided into two groups: positively and negatively correlated genes. The positively and negatively correlated genes were subsequently uploaded to query CMap, and searches were made for small-molecule compounds. Scores ranged from −1 to 1, representing the relationship between the drug and input genes, and drugs with a score of ≤−0.75 (*p* < 0.05, percent non-null >50) were considered candidate drugs.

Then, the candidate drugs were validated using the Comparative Toxicogenomics Database (CTD), which contains associations among chemicals, gene products and diseases (Davis et al., 2017).

## Results

### Aberrant Expression of NNMT in Patients With GC

**FIGURE 1:**
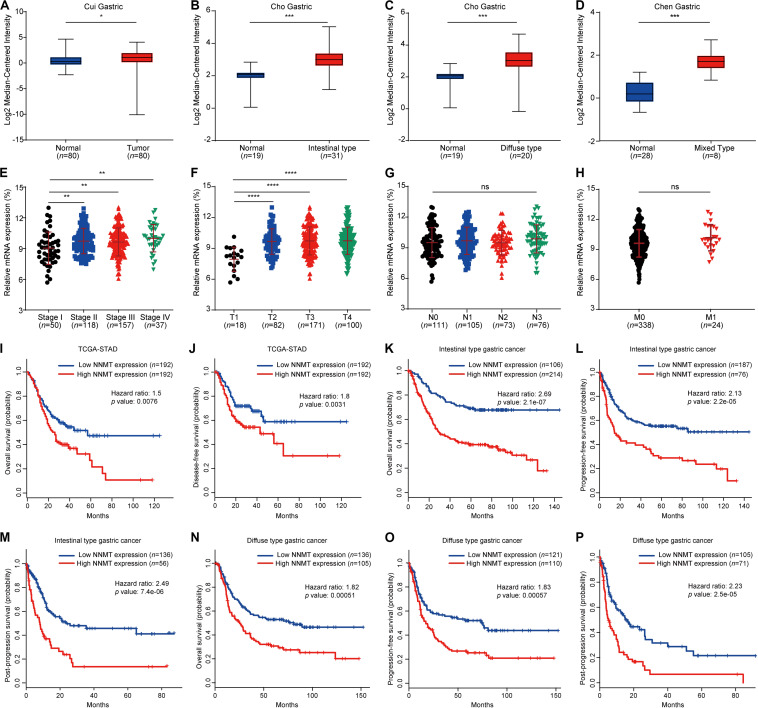
Differential expression of NNMT in different disease state and survival curves of differential NNMT expression. **(A–D)** The relative mRNA expression of NNMT in three Oncomine datasets: Cui Gastric **(A)**, Cho Gastric **(B,C)**, and Chen Gastric **(D)**. **(E–H)** The relative mRNA expression of NNMT in patients with different stages of gastric cancer in the LinkedOmics database. ns, no significance, *p < 0.05, **p < 0.01, ***p < 0.001, and ****p < 0.0001. **(I–J)** NNMT expression was associated with overall survival (OS) and disease-free survival (DFS) in STAD patients (GEPIA). **(K–M)** Kaplan-Meier curves of OS, FP, and PPS differences among intestinal-type gastric cancer patients (Kaplan-Meier plotter). **(N–P)** Kaplan-Meier curves of OS, FP and PPS differences among diffuse-type gastric cancer patients (Kaplan-Meier plotter).

First, we determined the relationships between NNMT expression and clinical GC development. NNMT expression in GC tissues was significantly higher than that in the paired normal tissues, according to Cui Gastric (Figure 1A), Cho Gastric (Figures 1B,C), and Chen Gastric (Figure 1D) data from the Oncomine database. In addition, increased NNMT was closely correlated with the stage of STAD (Figure 1E). Specifically, NNMT expression in STAD patients of stages T2-4 was significantly higher than that in T1-stage patients (Figure 1F). However, there was no significant difference in NNMT expression among STAD patients at different N and M stages (Figures 1G,H). These findings indicate an increasing trend for NNMT expression during malignant transformation in GC.

### The Prognostic Value of NNMT in Patients With GC

We further analyzed the relationship between NNMT expression and the survival of GC patients. Higher NNMT expression indicated a worse prognosis in STAD patients (Figures 1I,J). Specifically, patients with GC of the intestinal or diffuse type with higher NNMT expression had shorter OS, FP, and PPS (Figures 1K–P). In addition, a high level of NNMT was associated with a poor outcome in GC patients from stage II to IV and N1 to N3 (Table 1). However, overexpression of NNMT was remarkably correlated with better prognosis in HER2-negative GC patients who received 5-fluorouracil (5-FU)-based adjuvant treatment (Table 1), suggesting that NNMT might be a potential indicator of successful 5-FU therapy in HER2-negative GC patients.

**TABLE 1:** Correlation of NNMT expression and prognosis in gastric cancer with various clinicopathological factors.

Clinicopathological characteristics	Overall survival (*n* = 876)			Progression-free survival (*n* = 641)		
	*n*	Hazard ratio	*p*	*n*	Hazard ratio	*p*
**SEX**						
Female	236	1.47 (1.04−2.09)	**0.029**	201	1.58 (1.02−2.45)	**0.041**
Male	545	1.46 (1.16−1.83)	**0.00099**	438	1.37 (1.08−1.74)	**0.0096**
**STAGE**						
I	67	0.34 (0.12−0.98)	0.072	60	0.37 (0.11−1.22)	0.088
II	140	2.36 (1.09−5.11)	**0.024**	131	1.93 (1.06−3.52)	**0.029**
III	305	1.53 (1.15−2.03)	**0.0035**	186	1.84 (1.24−2.74)	**0.0022**
IV	148	2.00 (1.34−2.97)	**0.00049**	141	1.66 (1.11−2.44)	**0.01**
**STAGE T**						
2	241	2.05 (1.32−3.18)	**0.001**	239	1.97 (1.30−2.99)	**0.0012**
3	204	1.59 (1.12−2.27)	**0.0089**	204	1.31 (0.93−1.85)	0.12
4	38	1.90 (0.80−4.50)	0.14	39	2.50 (1.07−5.83)	**0.029**
**STAGE N**						
0	74	1.49 (0.64−3.45)	0.35	72	1.57 (0.68−3.63)	0.29
1	225	2.51 (1.65−3.84)	**9.8e**−**06**	222	2.28 (1.54−3.38)	**2.3e**−**05**
2	121	2.35 (1.44−3.82)	**0.00041**	125	2.28 (1.43−3.63)	**0.00037**
3	76	1.99 (1.16−3.42)	**0.011**	76	1.98 (1.16−3.38)	**0.01**
1 + 2 + 3	422	2.40 (1.82−3.16)	**1.6e**−**10**	423	2.16 (1.65−2.82)	**7.1e**−**09**
**STAGE M**						
0	444	1.96 (1.48−2.59)	**1.3e**−**06**	443	1.88 (1.44−2.45)	**2.3e**−**06**
1	56	2.46 (1.33−4.55)	**0.0033**	56	1.59 (0.86−2.94)	0.13
**LUREN CLASSIFICATION**						
Intestinal	320	2.69 (1.82−3.98)	**2.1e**−**07**	263	2.13 (1.49−3.05)	**2.2e**−**05**
Diffuse	241	1.82 (1.29−2.55)	**0.00051**	231	1.83 (1.29−2.58)	**0.00057**
**Treatment**						
Surgery alone	380	1.45 (1.09−1.94)	**0.011**	375	1.46 (1.10−1.93)	**0.0085**
5-FU-based adjuvant	153	0.55 (0.38−0.78)	**0.00075**	153	0.62 (0.43−0.90)	**0.012**
Other adjuvants	76	3.83 (1.58−9.27)	**0.0013**	80	4.10 (1.86−9.03)	**0.00015**
**HER2 status**						
negative	532	1.44 (1.15−1.80)	**0.0015**	408	1.47 (1.13−1.92)	**0.0038**
positive	344	1.40 (1.06−1.85)	**0.016**	233	1.42 (1.01−2.00)	**0.044**
**HER2 status (5-FU-based adjuvant)**						
HER2-negative	75	0.41 (0.23−0.71)	**0.001**	75	0.50 (0.28−0.88)	**0.014**
HER2-positive	78	0.64 (0.39−1.04)	0.07	78	0.71 (0.43−1.17)	0.18

*Data are from the probe 202238_s_at (NNMT), (*p* < 0.05) is in boldface.*

### PPI Network, Functional Annotation, and Pathway Enrichment Analysis of NNMT Correlated Genes in STAD Patients

**FIGURE 2:**
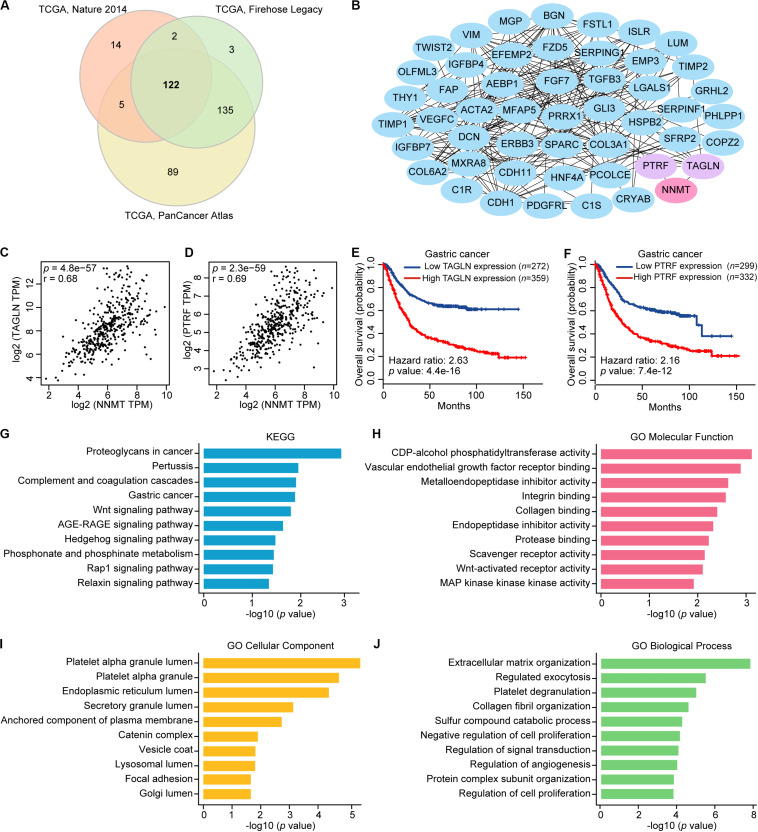
Identification, interaction analyses, and enrichment analysis of NNMT correlated genes in GC. **(A)** Overlap of genes correlated with NNMT expression in the three TCGA-STAD cohorts, including Nature 2014, Firehose Legacy, and PanCancer Atlas based on cBioPortal database. **(B)** The top 50 genes evaluated by connectivity degree in the PPI network of the 122 genes significantly correlated with NNMT expression. **(C)** Correlation between NNMT and TAGLN (*r* = 0.68). **(D)** Correlation between NNMT and PTRF (*r* = 0.69). **(E)** The prognostic values of TAGLN in gastric cancer patients (Kaplan-Meier plotter). **(F)** The prognostic values of PTRF in gastric cancer patients (Kaplan-Meier plotter). **(G)** Kyoto Encyclopedia of Genes and Genomes (KEGG) pathway enrichment of the 122 genes significantly correlated with NNMT expression. **(H–J)** Gene Ontology (GO) molecular function **(H)**, cellular components **(I)**, and biological processes **(J)** enriched for the 122 genes significantly correlated with NNMT expression.

Next, we identified NNMT correlated genes overlapped in the three TCGA-STAD cohorts (Figure 2A). The PPI network of these 122 genes was constructed using the STRING database. Next, the top 50 hub genes were screened out according to the node degree (Figure 2B). According to the PPI network, the TAGLN and PTRF genes can interact with NNMT. By using the GEPIA database, we found that these two genes were significantly correlated with NNMT expression (TAGLN: *r* = 0.68, PTRF: *r* = 0.69, both *p* < 0.0001) (Figures 2C,D). Consistently, the data from the K-M plotter database showed that TAGLN and PTRF mRNA levels were significantly related to the shorter OS of GC patients (*p* < 0.0001) (Figures 2E,F).

Then, we investigated the functions of NNMT correlated genes by using Enrichr. Figures 2G–J shows the top 10 most highly enriched Kyoto Encyclopedia of Genes and Genomes (KEGG) pathways and Gene Ontology (GO) items. Among the top 10 KEGG pathways, proteoglycans in cancer, gastric cancer, and Wnt signaling pathway were significantly related to the occurrence and development of GC. Also, the 122 genes were mapped onto GO for molecular function (MF), cellular component (CC), and biological process (BP) analyses. The top 10 most highly enriched MF category, CC category, and BP category included vascular endothelial growth factor receptor binding, integrin binding, Wnt-activated receptor activity, MAP kinase kinase kinase activity, focal adhesion, regulation of angiogenesis, and regulation of cell proliferation, which were significantly linked to the tumorigenesis and progression of GC.

### NNMT mRNA Levels Are Associated With Tumor-Infiltrating Immune Cells in Gastric Cancer

**FIGURE 3:**
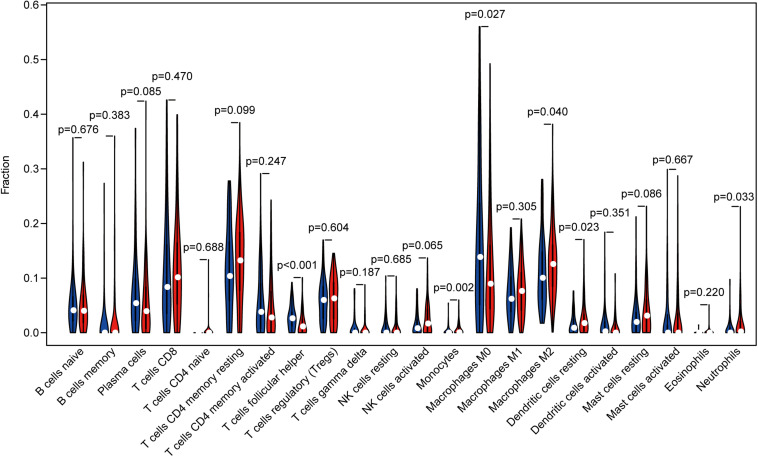
The proportion of 22 subpopulations of immune cells [T cells follicular helper, monocytes, M0 macrophages, M2 macrophages, resting dendritic cells, and neutrophils are main immune cells affected by NNMT expression. Among them, monocytes (*p* = 0.002), M2 macrophages (*p* = 0.040), resting dendritic cells (*p* = 0.023), and neutrophils (*p* = 0.033) are apparently increased in high expression group compared with low expression group. In contrast, T cells follicular helper (*p* < 0.001) and M0 macrophages (*p* = 0.027) are decreased in high expression group compared with low expression group].

Tumor infiltrating lymphocytes (TILs) is associated with sentinel lymph node status and clinical outcome in cancer patients (Azimi et al., 2012). In order to explore the relationship between NNMT expression and immune infiltration in STAD, we employed the CIBERSORT method to evaluate the gene expression profiles and the densities of 22 types of immune cells. Tumor samples were divided into two groups: NNMT high- and low-expression group. Among the 22 subpopulations of immune cells, T follicular helper cells (Tfh), monocytes, M0 macrophages, M2 macrophages, resting dendritic cells (DCs), and neutrophils were main immune cells affected by NNMT expression (Figure 3). Specifically, monocytes (*p* = 0.002), M2 macrophages (*p* = 0.040), resting DCs (*p* = 0.023), and neutrophils (*p* = 0.033) are apparently increased in NNMT high-expression group compared with low-expression group. Conversely, Tfh (*p* < 0.001) and M0 macrophages (*p* = 0.027) are decreased in NNMT high-expression group compared with low-expression group.

**FIGURE 4:**
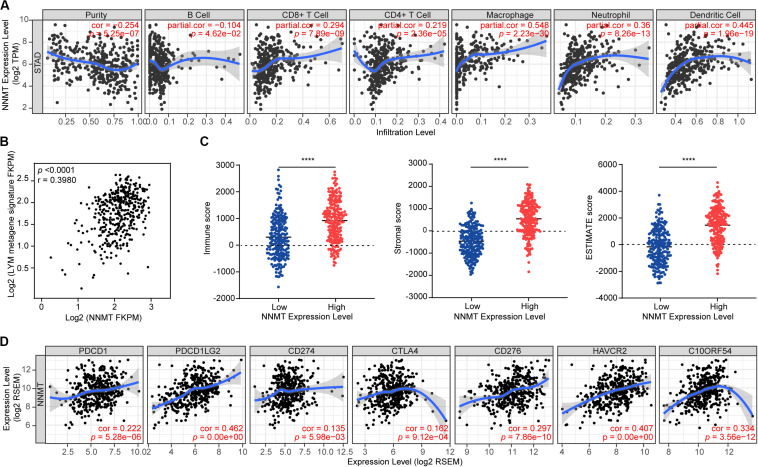
Correlation of NNMT expression with immune infiltration level in STAD. **(A)** NNMT expression is negatively correlated with tumor purity and infiltrating level of B cells (*r* = –0.104, *p* = 4.62e–02), and has significant positive correlations with infiltrating levels of CD8 + T cells (*r* = 0.294, *p* = 7.89e–09), CD4 + T cells (*r* = 0.219, *p* = 2.36e–05), macrophages (*r* = 0.548, *p* = 2.23e–30), neutrophils (*r* = 0.36, *p* = 8.26e–13), and dendritic cells (*r* = 0.445, *p* = 1.96e–19) in STAD (*n* = 415). **(B)** The average expression of the LYM metagene signature in each GC patient from TCGA is shown relative to that for NNMT mRNA. **(C)** NNMT mRNA levels are shown relative to levels of immune, stromal, and ESTIMATE gene signatures calculated by the ESTIMATE algorithm, ****p < 0.0001. **(D)** NNMT expression correlated with the checkpoint molecules in STAD. Markers include PDCD1 (PD1), PDCD1LG2 (PDL2), CD274 (PDL1), CTLA4, CD276 (B7-H3), HAVCR2 (TIM-3), and C10ORF54 (VISITA).

Then, we used the TIMER to further analyze the association between NNMT expression and immune infiltration levels in STAD. Tumor purity was determined by evaluating the immune infiltration into tumor tissues by means of genomic methods (Yoshihara et al., 2013). We found that NNMT expression was significantly negatively associated with the infiltration level of B cells (*r* = −0.104, *p* = 4.62e−02), while significantly positively related to the infiltration levels of CD8 + T cells (*r* = 0.294, *p* = 7.89e−09), CD4 + T cells (*r* = 0.219, *p* = 2.36e−05), macrophages (*r* = 0.548, *p* = 2.23e−30), neutrophils (*r* = 0.36, *p* = 8.26e−13), and DCs (*r* = 0.445, *p* = 1.96e−19) (Figure 4A), indicating that NNMT promotes immune infiltration in GC.

### Analysis of the Association Between NNMT Expression and Immune Signature in Gastric Cancer

Moreover, a LYM metagene signature is referred as tumor infiltration by lymphocytes and is associated with good prognosis in breast cancer (Cheng et al., 2013). In order to investigate the relationship between NNMT and the LYM metagene in GC, we performed the correlation analysis of NNMT mRNA levels and the T-cell transcript-enriched LYM metagene signature. As shown in Figure 4B, there was a significant correlation between NNMT overexpression and the LYM signature in STAD (*r* = 0.3980, *p* < 0.0001). To confirm the correlation, we applied the ESTIMATE algorithm to calculate the immune score, stromal score, and ESTIMATE score in STAD. When we divided the top 50% of the patients into an NNMT high-expression group (*n* = 208), and the bottom 50% into a low-expression group (*n* = 207) according to their individual expression levels of NNMT, we found that the high NNMT group exhibited higher levels of immune score, stromal score, and ESTIMATE score than did the low NNMT group. Collectively, these data indicate that increased NNMT is associated with tumor immune infiltration in GC.

In order to explore the value of NNMT in guiding treatment decisions, we screened for genes enriched in immune checkpoint pathways in STAD. Accordingly, we identified the following genes as being positively correlated with NNMT expression (Figure 4C): PDCD1 (PD1), PDCD1LG2 (PDL2), CD274 (PDL1), CTLA4, CD276 (B7-H3), HAVCR2 (TIM-3), and C10ORF54 (VISITA), suggesting that patients with high NNMT expression may have favorable responses to therapies involving immune checkpoint blockade.

### Prediction of Potential Therapeutic Drugs for Gastric Cancer

Given the association of NNMT expression with both the immune infiltration level and outcome in GC, we postulated that drugs affecting the expression of NNMT and its correlated genes might play an antitumor role in GC treatment. NNMT and its correlated genes were regarded as input files for the CMap pilot dataset. As a result, 19 compounds (Mean ≤ −0.4, *p* < 0.01, percent non-null >50) were considered candidate drugs (Table 2). Then, the relationships between the 19 compounds and cancers were investigated using CTD, and the results revealed that 10 drugs, namely, the β adrenal receptor blocker nadolol; the antifibrinolytic hemostatic tranexamic acid; the antispasmodic drug adiphenine; the antiseptic agent chlorhexidine; the antiemetic trimethobenzamide; the histamine H1 antagonist clemastine; the non-steroidal anti-inflammatory drug felbinac; the class Ia antiarrhythmic agent ajmaline; the imidazole antithyroid agent carbimazole; and the antibiotic dapsone, as potential drugs for cancer treatment (Table 3). Finally, according to these results, nadolol, tranexamic acid, felbinac and dapsone were considered as promising drugs for GC (Supplementary Table 1).

**TABLE 2:** The small molecules that can reverse the expression of NNMT and its correlated genes after CMap analysis.

CMap name	Mean	*n*	Enrichment	*p*	Specificity	Percent non-null	Biological function
Prestwick-692	−0.669	4	−0.918	0.0001	0	100	
Nadolol	−0.657	4	−0.904	0.00016	0	100	β adrenal receptor blocker
Isoflupredone	−0.705	3	−0.931	0.0005	0.025	100	
Tranexamic acid	−0.577	5	−0.796	0.00074	0.0201	100	Antifibrinolytic hemostatic
Etiocholanolone	−0.47	6	−0.724	0.00085	0.039	100	
Pheneticillin	−0.562	4	−0.851	0.00092	0.0065	100	Antibiotic
Midodrine	−0.458	5	−0.74	0.00242	0.0451	100	Adrenergic alpha agonist
Heptaminol	−0.446	5	−0.715	0.00411	0.0343	80	Cardiac stimulant
Adiphenine	−0.596	5	−0.707	0.00469	0.2177	80	Antispasmodic drug
Chlorhexidine	−0.433	5	−0.701	0.00515	0.015	80	Antiseptic agent
Trimethobenzamide	−0.508	5	−0.694	0.00583	0.047	80	Antiemetic
Clemastine	−0.613	3	−0.846	0.00735	0.0336	100	Histamine H1 antagonist
Felbinac	−0.506	4	−0.744	0.00849	0.1277	100	Non-steroidal anti-inflammatory drug
Prestwick−1082	−0.552	3	−0.837	0.00857	0.0984	100	
Ajmaline	−0.662	3	−0.836	0.00877	0.0284	100	Class Ia antiarrhythmic agent
Carbimazole	−0.623	3	−0.835	0.00891	0.0206	100	Imidazole antithyroid agent
Dicycloverine	−0.404	5	−0.668	0.00939	0.0382	80	Muscarinic receptor antagonist
Prestwick-642	−0.502	4	−0.738	0.00941	0.0414	100	
Dapsone	−0.403	5	−0.666	0.00987	0.0534	80	Antibiotic

**TABLE 3:** Relationship between cancers and eight potential drugs based on CTD.

Drugs	Disease	Inference Score
Nadolol	Stomach Neoplasms	5.67
	Gastro-enteropancreatic neuroendocrine tumor	4.58
	Prostatic Neoplasms	4.43
	Leukemia-Lymphoma, Adult T-Cell	4.14
	Mesothelioma, Malignant	3.95
	Glioma	3.83
	Colonic Neoplasms	3.7
	Neoplasms, Experimental	3.42
	Carcinoma, Hepatocellular	3.38
	Carcinoma, Non-Small-Cell Lung	3.31
	Liver Neoplasms	3.31
	Mammary Neoplasms, Experimental	3.18
	Lung Neoplasms	2.90
	Breast Neoplasms	2.71
Adiphenine	Barrett Esophagus	5.74
	Osteosarcoma	5.13
	Esophageal Neoplasms	4.81
	Adenocarcinoma	4.44
Chlorhexidine	Hematologic Neoplasms	4.38
	Leukemia, Myelogenous, Chronic, BCR-ABL Positive	4.02
	Esophageal Squamous Cell Carcinoma	3.75
	Carcinoma, Hepatocellular	2.89
Trimethobenzamide	Barrett Esophagus	5.28
	Osteosarcoma	4.67
	Esophageal Neoplasms	4.35
	Adenocarcinoma	3.99
Felbinac	Thyroid Neoplasms	3.69
	Glioblastoma	3.59
	Adenocarcinoma	3.25
	Colonic Neoplasms	3.18
	Neoplasm Invasiveness	3.12
	Stomach Neoplasms	3.07
	Neoplasms	3.06
	Lung Neoplasms	2.94
	Breast Neoplasms	2.71
Clemastine	Carcinoma, Ovarian Epithelial	†
	Colorectal Neoplasms	3.94
	Cell Transformation, Neoplastic	3.72
	Colonic Neoplasms	3.70
	Carcinoma, Hepatocellular	3.60
	Neoplasms	3.58
Ajmaline	Osteosarcoma	3.52
	Cell Transformation, Neoplastic	3.17
	Colonic Neoplasms	3.15
	Neoplasms	3.05
	Prostatic Neoplasms	2.50
	Breast Neoplasms	2.47
Carbimazole	Colonic Neoplasms	3.56

*†, therapeutic value.*

## Discussion

In the present study, we first confirmed that NNMT is overexpressed and associated with a poor outcome in GC. Nevertheless, our results also showed that a high level of NNMT was related to longer survival in HER2-negative GC patients who received 5-FU-based adjuvant chemotherapy, suggesting that NNMT could be an indicator of successful 5-FU treatment in GC.

Then, we explored the interactions among NNMT and its correlated genes, and found that TAGLN and PTRF can interact with NNMT. Overexpression of the TAGLN and PTRF genes was significantly associated with a worse prognosis in GC patients. Therefore, TAGLN and PTRF may exert tumorigenic effects on NNMT to promote GC progression. We found that NNMT and its correlated genes are mainly related to the tumorigenesis and development of GC, suggesting that NNMT serve a vital role in the development of GC and could be a therapeutic target.

Another important part of this study was our finding that NNMT expression was correlated with immune infiltration in GC. According to the CIBERSORT algorithm method, there were positive correlations between NNMT expression and infiltration levels of monocytes, M2 macrophages, resting DCs, and neutrophils. A prior study has demonstrated that higher infiltration of M2 macrophages was significantly associated with worse prognosis in esophageal cancer (Yamamoto et al., 2020). Furthermore, previous studies have revealed that tumor-infiltrating monocytes and macrophages could promote tumor invasion and migration by upregulating S100A8 and S100A9 expression in cancer cells (Lim et al., 2016). As for tumor-infiltrating DCs (TIDCs), the complexities of their role are not fully understood, but it is evident that the functions of TIDCs are weakened in many aspects, which lead TIDCs to suppress immune system rather than activate immune system in the tumor microenvironment (Tran Janco et al., 2015). As for neutrophils, it is suggested that they are potent drivers of tumor angiogenesis (Mantovani et al., 2011). More recently, it is reported that GC patients with higher level of tumor-infiltrating neutrophils were prone to benefit from postoperative adjuvant chemotherapy and had prolonged survival time (Zhang et al., 2018). These findings may aid in explaining GC patients with high NNMT expression exhibited unfavorable outcome compared to those with low expression of NNMT.

On the other side, there was a negative correlation between NNMT expression and infiltration levels of Tfh and M0 macrophages. Tfh have been shown to predict longer survival in breast cancer patients (Gu-Trantien et al., 2013), as they are critical in germinal center formation, affinity maturation, and helping B cells produce antibody (Crotty, 2014). Additionally, M0 macrophages are originated from monocytes and are not yet polarized to either the M1 or M2 macrophages. Previous studies have reported that M0 macrophages are correlated with decreased survival time in estrogen receptor-positive breast cancer patients (Bense et al., 2017) and hepatocellular cancer patients (Hsiao et al., 2019). Therefore, the impacts of tumor-infiltrating M0 macrophages on the prognosis of GC patients need further research and summarization.

According to TIMER database, there were significant positive correlations between the NNMT expression levels and infiltration levels of CD8 + and CD4 + T cells, macrophages, neutrophils, and DCs in STAD. TILs are comprised primarily of CD8 + and CD4 + T cells (Pruneri et al., 2018), and higher CD8 + T cells infiltration levels were associated with more favorable overall response rates in breast cancer (Salgado et al., 2015; Savas et al., 2016). However, accumulating evidence have indicated that the antitumor immune efficacy of tumor-infiltrating CD8 + T cells is likely to be affected by immunosuppressive molecules in tumor microenvironment (Canale et al., 2018) and their distribution in tumor tissue. For example, the high density of CD8 + cell in the tumor center predicts prolonged survival, but high density of CD8 + cell in the tumor margin dose not. Generally, density of CD8 + cell has extensive heterogeneity, and the CD8 + cell density in the tumor center was less than half that in the tumor border (Masugi et al., 2019). On the other hand, there was a significant negative correlation between the NNMT expression and infiltration level of B cells. High density of tumor-infiltrating B cells has reported to improve OS in patients with squamous cell carcinomas and adenocarcinoma of the esophagogastric junction (Knief et al., 2016; Kim et al., 2020), while B cells could promote cellular immunity by presenting antigen, shaping tertiary lymphoid structures and producing cytokines (Nielsen and Nelson, 2012). Thus, the negative influence of NNMT on outcome of GC may due to the decrease of tumor-infiltrating B cells.

Furthermore, high NNMT expression was associated with increased Immunoscore in STAD. As Immunoscore is a routine parameter in predicting the response to immunotherapy (Galon and Bruni, 2019), NNMT has potential as a parameter for making treatment decisions. To be precise, NNMT showed significant correlations with the expression of immune checkpoint markers such as PD1, PDL1, PDL2, and CTLA4. Strategies used to target CTLA4 and PD-1 and/or PD-L1 have now been approved by the US Food and Drug Administration for the treatment of multiple cancers (Herbst et al., 2014; Davoli et al., 2017). Blockade of CSF1R could promote antitumor cytotoxic T-cell response following PD-L1 blockade by diminishing the pro-tumoral macrophage population (Cannarile et al., 2017). Taken together, our findings show that NNMT is an indicator of “hot” tumors (highly infiltrated, with high immune cells densities in both locations) and could help guide immunotherapy.

Previous studies have shown that NNMT is a potential marker in some cancers (Lu and Long, 2018), few studies have focused on the biological role of NNMT. So far, Seta et al. found that overexpression of NNMT was associated with upregulation of an anti-apoptotic protein survivin-ΔEx3 in OSCC cell line (HSC-2) (Seta et al., 2019), and Akar et al. reported that NNMT overexpression was correlated with aberrant p53 and phospho-Akt expression in high-grade endometrial cancer (Akar et al., 2019).

NNMT was reported to regulate methylation of tumor-associated genes. For example, Li et al. demonstrated that NNMT promoted hepatocellular carcinoma cells invasion and metastasis by modifying the histone H3 methylation and regulating cluster of differentiation 44 (CD44) (Li et al., 2019). And Ulanovskaya showed that NNMT altered the protein methylation state of tumor cells through regulating cellular SAM/SAH ratios (Ulanovskaya et al., 2013). Similarly, Jung et al. raised that elevated NNMT levels caused DNA hypomethylation by reducing a key upstream methyl group donor methionine, thus shifting tumors toward a mesenchymal phenotype and accelerating tumor growth (Jung et al., 2017).

NNMT was indicated to affect susceptibility to some anticancer drugs. Specifically, Parsons et al. observed that NNMT expression and its product 1-methylnicotinamide could protected SH-SY5Y neuroblastoma cells from the toxicity of the Complex I inhibitors MPP + (1-methyl-4-phenylpyridinium ion) and rotenone (Parsons et al., 2011), and Wang et al. showed that ectopic overexpression of NNMT significantly decreased the cancer-killing effects of adriamycin and paclitaxel by stabilizing SIRT1 protein in breast cancer cells (Wang et al., 2019). Interestingly, BRCA1 depletion-induced NNMT upregulation was shown to promote metabolic reprogramming of ovarian cancer cells, which sensitized ovarian cancer cells to agents that inhibit mitochondrial metabolism (VLX600 and tigecycline) and to agents that inhibit glucose import (WZB117) (Kanakkanthara et al., 2019).

Notably, we identified 19 small-molecule drugs that could potentially reverse the gene expression patterns that are accompanied by overexpression of NNMT in GC. Among these drugs, further analysis showed that nadolol, tranexamic acid, felbinac and dapsone are the four most promising drugs in treating GC. To date, no studies have clarified the effects of tranexamic acid and felbinac in GC. Nadolol, a non-selective beta adrenal receptor blocker, is used to lower blood pressure. Previous studies have not investigated the effects of nadolol in GC, but other beta-adrenoceptor antagonists have been studied in GC. As role of adrenergic receptors in gastric tumor growth has been recognized in recent years (Shin et al., 2008), propranolol, another non-selective-adrenergic antagonist, has been shown to inhibit cell proliferation and induce apoptosis in gastric cancer cell lines (SGC-7901 and BGC-823) (Liao et al., 2010). Furthermore, beta-adrenoceptor antagonists have been shown to reverse the stimulatory action of nicotine on the expression of protein kinase C, extracellular signal-regulated kinase-1/2 phosphorylation, and cyclooxygenase 2 together with cell proliferation in gastric cancer cell line (AGS) (Shin et al., 2007). More recently, additional studies have indicated that propranolol could enhance the sensitivity of gastric cancer cells to radiotherapy by inhibiting beta-adrenoceptors, NF-κB expression, and its downstream genes: VEGF, EGFR, and COX-2 (Liao et al., 2010, 2018). Dapsone, an aniline derivative belonging to the group of synthetic sulfones, has antimicrobial/antiprotozoal effects and anti-inflammatory features similarly to non-steroidal anti-inflammatory drugs. In recent years, some dapsone derivatives have been proved to have anticancer activity (Park et al., 2011; Wozel and Blasum, 2014) in breast cancer (Al-Said et al., 2012) and glioma (Hargrave, 2009), as acyl derivatives of dapsone might inhibit the arginine methyltransferase hPRMT1 (Bissinger et al., 2011). Nevertheless, the antitumor effects of dapsone and its derivatives in GC require further clarification, and the proposed mechanisms leave room for speculations. Therefore, future experiments are needed to verify the results.

In summary, high expression of NNMT in GC is a predictor of poor outcome. Given its important role in immune cell infiltration, NNMT can also serve as a biomarker for immunotherapy. Moreover, nadolol, tranexamic acid, felbinac, and dapsone may be reasonable drug candidates for the treatment of GC.

## Data Availability Statement

All datasets generated for this study are included in the article/Supplementary Material.

## Author Contributions

MW, WH, and GW designed the study. MW, WH, and YY carried out data acquisition and analysis. MW, WH, GW, and YY wrote the manuscript. X-FY supervised the study, participated in its design, interpretation, and analysis, and including drafting. All authors read and approved the final manuscript.

## Conflict of Interest

The authors declare that the research was conducted in the absence of any commercial or financial relationships that could be construed as a potential conflict of interest.

## Footnotes

1 www.oncomine.org

2 www.kmplot.com

3 http://cbioportal.org

4 http://www.linkedomics.org

5 http://string-db.org/

6 https://bioinformatics.mdanderson.org/estimate/

7 http://cibersort.stanford.edu/

8 https://cistrome.shinyapps.io/timer/
