# Supplementary material for: Nicotinamide N-Methyltransferase Is a Prognostic Biomarker and Correlated With Immune Infiltrates in Gastric Cancer
Source: Front Genet. 2020 Oct 28;11:580299. doi: 10.3389/fgene.2020.580299 (PMC7655872; doi:10.3389/fgene.2020.580299)
Supplement: Supplementary file 1 [file Table_1.DOC]

**TABLE S1** Relationship between cancers and eight potential drugs based on CTD

| Drugs | Disease | Inference Score |
| --- | --- | --- |
| Nadolol | Stomach Neoplasms | 5.67 |
|  | Gastro-enteropancreatic neuroendocrine tumor | 4.58 |
|  | Prostatic Neoplasms | 4.43 |
|  | Leukemia-Lymphoma, Adult T-Cell | 4.14 |
|  | Mesothelioma, Malignant | 3.95 |
|  | Glioma | 3.83 |
|  | Colonic Neoplasms | 3.7 |
|  | Neoplasms, Experimental | 3.42 |
|  | Carcinoma, Hepatocellular | 3.38 |
|  | Carcinoma, Non-Small-Cell Lung | 3.31 |
|  | Liver Neoplasms | 3.31 |
|  | Mammary Neoplasms, Experimental | 3.18 |
|  | Lung Neoplasms | 2.9 |
|  | Breast Neoplasms | 2.71 |
| Tranexamic Acid | Leiomyoma | † |
|  | Uterine Neoplasms | † |
|  | Mesothelioma, Malignant | 9.29 |
|  | Intestinal Neoplasms | 6.8 |
|  | Prostatic Neoplasms | 5.68 |
|  | Multiple Myeloma | 5.55 |
|  | Esophageal Neoplasms | 5.47 |
|  | Polycystic Ovary Syndrome | 5.14 |
|  | AIDS-related Kaposi sarcoma | 4.91 |
|  | Adenocarcinoma, Clear Cell | 4.57 |
|  | Cell Transformation, Neoplastic | 4.55 |
|  | Neoplasm Invasiveness | 4.5 |
|  | Chondrosarcoma, Mesenchymal | 4.46 |
|  | Colonic Neoplasms | 4.16 |
|  | Colorectal Neoplasms | 4.13 |
|  | Sarcoma, Synovial | 3.94 |
|  | Soft Tissue Neoplasms | 3.94 |
|  | Esophageal Squamous Cell Carcinoma | 3.88 |
|  | Breast Neoplasms | 3.87 |
|  | Gallbladder Neoplasms | 3.7 |
|  | Lymphoma, T-Cell, Cutaneous | 3.57 |
|  | Lymphoma, Follicular | 3.54 |
|  | Precursor T-Cell Lymphoblastic Leukemia-Lymphoma | 3.27 |
|  | Prostate cancer, familial | 3.23 |
|  | Head and Neck Neoplasms | 3.19 |
|  | Lymphoma, Large B-Cell, Diffuse | 3.16 |
|  | Leukemia, Lymphocytic, Chronic, B-Cell | 3.14 |
|  | Precursor Cell Lymphoblastic Leukemia-Lymphoma | 2.85 |
|  | Lymphoma, Non-Hodgkin | 2.82 |
|  | Adenoma | 2.74 |
|  | Leukemia, Myeloid, Acute | 2.6 |
|  | Melanoma | 2.59 |
|  | Adenocarcinoma | 2.51 |
|  | Carcinoma | 2.51 |
|  | Liver Neoplasms, Experimental | 2.41 |
|  | Carcinoma, Hepatocellular | 2.38 |
| Adiphenine | Barrett Esophagus | 5.74 |
|  | Osteosarcoma | 5.13 |
|  | Esophageal Neoplasms | 4.81 |
|  | Adenocarcinoma | 4.44 |
| Chlorhexidine | Hematologic Neoplasms | 4.38 |
|  | Leukemia, Myelogenous, Chronic, BCR-ABL Positive | 4.02 |
|  | Esophageal Squamous Cell Carcinoma | 3.75 |
|  | Carcinoma, Hepatocellular | 2.89 |
| Trimethobenzamide | Barrett Esophagus | 5.28 |
|  | Osteosarcoma | 4.67 |
|  | Esophageal Neoplasms | 4.35 |
|  | Adenocarcinoma | 3.99 |
| Clemastine | Carcinoma, Ovarian Epithelial | † |
|  | Colorectal Neoplasms | 3.94 |
|  | Cell Transformation, Neoplastic | 3.72 |
|  | Colonic Neoplasms | 3.7 |
|  | Carcinoma, Hepatocellular | 3.6 |
|  | Neoplasms | 3.58 |
| Felbinac | Thyroid Neoplasms | 3.69 |
|  | Glioblastoma | 3.59 |
|  | Adenocarcinoma | 3.25 |
|  | Colonic Neoplasms | 3.18 |
|  | Neoplasm Invasiveness | 3.12 |
|  | Stomach Neoplasms | 3.07 |
|  | Neoplasms | 3.06 |
|  | Lung Neoplasms | 2.94 |
|  | Breast Neoplasms | 2.71 |
| Ajmaline | Osteosarcoma | 3.52 |
|  | Cell Transformation, Neoplastic | 3.17 |
|  | Colonic Neoplasms | 3.15 |
|  | Neoplasms | 3.05 |
|  | Prostatic Neoplasms | 2.5 |
|  | Breast Neoplasms | 2.47 |
| Carbimazole | Colonic Neoplasms | 3.56 |
| Dapsone | Prostatic Neoplasms | † |
|  | Breast Neoplasms | † |
|  | Cell Transformation, Neoplastic | 18.19 |
|  | Neoplasm Invasiveness | 16.74 |
|  | Carcinoma, Hepatocellular | 15.67 |
|  | Neoplasm Metastasis | 13.63 |
|  | Mesothelioma, Malignant | 12.89 |
|  | Carcinoma, Squamous Cell | 12.42 |
|  | Kidney Neoplasms | 11.53 |
|  | Brain Neoplasms | 10.85 |
|  | Urinary Bladder Neoplasms | 10.54 |
|  | Colonic Neoplasms | 10.09 |
|  | Skin Neoplasms | 9.88 |
|  | Carcinoma, Non-Small-Cell Lung | 9.82 |
|  | Colorectal Neoplasms | 9.81 |
|  | Pancreatic Neoplasms | 9.75 |
|  | Lung Neoplasms | 9.31 |
|  | Glioblastoma | 8.88 |
|  | Stomach Neoplasms | 8.84 |
|  | Adenocarcinoma | 8.5 |
|  | Mesothelioma | 7.61 |
|  | Barrett Esophagus | 7.17 |
|  | Ovarian Neoplasms | 7.08 |
|  | Leukemia-Lymphoma, Adult T-Cell | 6.37 |
|  | Leukemia, Myelogenous, Chronic, BCR-ABL Positive | 6 |
|  | Carcinoma, Renal Cell | 5.78 |
|  | Squamous Cell Carcinoma of Head and Neck | 5.55 |
|  | Sarcoma | 5.53 |
|  | Liver Neoplasms, Experimental | 5.48 |
|  | Lymphoma, Large B-Cell, Diffuse | 5.42 |
|  | Head and Neck Neoplasms | 5.38 |
|  | Osteosarcoma | 5.36 |
|  | Hodgkin Disease | 5.05 |
|  | Neoplasms | 5.05 |
|  | Neoplasm, Residual | 4.98 |
|  | Esophageal Squamous Cell Carcinoma | 4.9 |
|  | Choledochal Cyst | 4.89 |
|  | Precursor Cell Lymphoblastic Leukemia-Lymphoma | 4.88 |
|  | Endometrial Neoplasms | 4.87 |
|  | Papilloma | 4.85 |
|  | Precancerous Conditions | 4.76 |
|  | Neoplasm Regression, Spontaneous | 4.72 |
|  | Tuberous Sclerosis 2 | 4.7 |
|  | Carcinoma | 4.69 |
|  | Lymphomatoid Papulosis | 4.52 |
|  | Thyroid Carcinoma, Anaplastic | 4.52 |
|  | Thyroid Neoplasms | 4.49 |
|  | Lymphoma, Large B-Cell, Diffuse | 4.42 |
|  | Gliosarcoma | 4.33 |
|  | Glioma | 4.31 |
|  | Mesothelioma | 4.27 |
|  | Neoplasms, Experimental | 4.19 |
|  | Salivary Gland Neoplasms | 4.19 |
|  | Carcinosarcoma | 4.18 |
|  | Pancreatic Carcinoma | 4.1 |
|  | Tuberous Sclerosis | 4.08 |
|  | Glioma | 4.03 |
|  | Hamartoma | 4.03 |
|  | Vaginal Neoplasms | 3.95 |
|  | Adrenocortical Carcinoma | 3.94 |
|  | Carcinoma, Non-Small-Cell Lung | 3.83 |
|  | Precancerous Conditions | 3.71 |
|  | Lymphoma, Large-Cell, Anaplastic | 3.69 |
|  | Prostatic Intraepithelial Neoplasia | 3.68 |
|  | Cell Transformation, Neoplastic | 3.63 |
|  | Neoplasm Invasiveness | 3.55 |
|  | Neoplasm Metastasis | 3.51 |
|  | Neoplasms | 3.49 |
|  | Bone Neoplasms | 3.47 |
|  | Carcinoma, Ductal, Breast | 3.44 |
|  | Tongue Neoplasms | 3.43 |
|  | Mammary Neoplasms, Experimental | 3.4 |
|  | Nasopharyngeal Carcinoma | 3.39 |
|  | Adenomatous Polyposis Coli | 3.35 |
|  | Lymphatic Metastasis | 3.35 |
|  | Liver Neoplasms | 3.27 |
|  | Melanoma | 3.25 |
|  | Astrocytoma | 3.24 |
|  | Sezary Syndrome | 3.21 |
|  | Breast Neoplasms | 3.12 |
|  | Leukemia | 2.92 |
|  | Leukemia, Promyelocytic, Acute | 2.88 |
|  | Mouth Neoplasms | 2.86 |
|  | Multiple Myeloma | 2.71 |
|  | Esophageal Neoplasms | 2.7 |
|  | Adenocarcinoma of Lung | 2.61 |
| †, proven therapeutic value. | |  |
